# Epileptogenic networks in extra temporal lobe epilepsy

**DOI:** 10.1162/netn_a_00327

**Published:** 2023-12-22

**Authors:** Gerard R. Hall, Frances Hutchings, Jonathan Horsley, Callum M. Simpson, Yujiang Wang, Jane de Tisi, Anna Miserocchi, Andrew W. McEvoy, Sjoerd B. Vos, Gavin P. Winston, John S. Duncan, Peter N. Taylor

**Affiliations:** CNNP Lab, Interdisciplinary Computing and Complex BioSystems Group, School of Computing, Newcastle University, Newcastle upon Tyne, United Kingdom; Department of Epilepsy, UCL Queen Square Institute of Neurology, London, United Kingdom; Faculty of Medical Sciences, Newcastle University, Newcastle upon Tyne, United Kingdom; UCL/UCLH NIHR University College London Hospitals Biomedical Research Centre, London, United Kingdom; Centre for Microscopy, Characterisation, and Analysis, University of Western Australia, Nedlands, Australia; Department of Medicine, Division of Neurology, Queen’s University, Kingston, Canada

**Keywords:** Extra temporal lobe epilepsy, Diffusion MRI, Epileptogenic zone, Structural connectivity, Resection, Seizure, Tractography, Connectome, Network

## Abstract

Extra temporal lobe epilepsy (eTLE) may involve heterogenous widespread cerebral networks. We investigated the structural network of an eTLE cohort, at the postulated epileptogenic zone later surgically removed, as a network node: the resection zone (RZ). We hypothesized patients with an abnormal connection to/from the RZ to have proportionally increased abnormalities based on topological proximity to the RZ, in addition to poorer post-operative seizure outcome. Structural and diffusion MRI were collected for 22 eTLE patients pre- and post-surgery, and for 29 healthy controls. The structural connectivity of the RZ prior to surgery, measured via generalized fractional anisotropy (gFA), was compared with healthy controls. Abnormal connections were identified as those with substantially reduced gFA (*z* < −1.96). For patients with one or more abnormal connections to/from the RZ, connections with closer topological distance to the RZ had higher proportion of abnormalities. The minority of the seizure-free patients (3/11) had one or more abnormal connections, while most non-seizure-free patients (8/11) had abnormal connections to the RZ. Our data suggest that eTLE patients with one or more abnormal structural connections to/from the RZ had more proportional abnormal connections based on topological distance to the RZ and associated with reduced chance of seizure freedom post-surgery.

## Author Summary

Focal epilepsy describes seizures that originate at one or more location. A significant proportion of cases, however, are unresponsive to drug treatment; therefore, surgery remains an important and effective treatment in stopping seizures. We wanted to further understand the nature of these networks associated with epilepsy and how they contribute to post-surgical outcome. Our research asked three questions: Does distance to the clinically suspected site of the epilepsy increase the likelihood of network abnormality? Can an upstream abnormal connection to a region increase the chance of abnormal connections downstream? Can the presence of abnormal connections explain post-operative outcome in terms of seizure freedom? We hope our work will shed light on epileptogenic networks and potentially stimulate new ideas and discussions to investigate brain networks in epilepsy.

## INTRODUCTION

Epilepsy is a major neurological condition affecting over 50 million people worldwide. Characterized by recurring spontaneous seizures, with transient changes in awareness and/or behavior, epilepsy can lead to long-term cognitive, neurobiological, psychosocial morbidity, and premature mortality (Fisher et al., 2014). Focal epilepsy accounts for 60% of adult epilepsy and describes seizures as originating from a particular location in the brain, termed the seizure onset zone (SOZ) (Tellez-Zenteno & Hernandez-Ronquillo, 2012). Epilepsy surgery involves the resection of the area thought to be involved in generating seizures, with the subsequent resected area termed the resection zone (RZ). Temporal lobe epilepsy (TLE) resections are most common, with the remaining third of focal epilepsies arising outside the temporal lobe (Chowdhury, Silva, Whatley, & Walker, 2021; Delev et al., 2019). Extratemporal lobe epilepsy (eTLE) may arise from numerous sites in the brain and, inevitably, cohorts are heterogenous.

Imaging modalities have provided valuable insights into eTLE, from analyzing the structure of pathology to help classify and understand eTLE subtypes (Hong, Bernhardt, Schrader, Bernasconi, & Bernasconi, 2016; Wagstyl et al., 2022), to reporting alterations in widespread functional networks across the brain (Carboni et al., 2020; Hong et al., 2019; Kini et al., 2019; Ridley et al., 2015), to identifying distant structural networks that result in long-term seizure freedom if disconnected (Giampiccolo et al., 2023). Diffusion MRI (dMRI) has been important in understanding the role of structural networks in epilepsy. Focal epilepsies affect a widespread network rather than a localized zone (Englot, Konrad, & Morgan, 2016), from specific connections (Giampiccolo et al., 2023) to widespread global changes (Tavakol et al., 2019). A recent large-scale multicenter study (21 cohorts, 1,249 total patients) reported widespread reductions in dMRI measures of fractional anisotropy (FA) in 36 of 38 major connective tracts when comparing all epilepsy types against controls (Hatton et al., 2020). Changes in connective tracts were also present in TLE and eTLE with no visual MRI pathology. Left and right TLE were associated with lower FA in 20 and 19 connective regions, respectively; those with eTLE had lower FA in 33 connective regions, compared with controls. These findings support the importance of understanding network mechanisms in focal epilepsy and suggest that connectivity changes are widespread, in eTLE and TLE.

Connectivity abnormalities in TLE are better understood than in eTLE. In TLE, specific changes in white matter connections have been identified and reproduced across studies, and in some cases have been shown to be widespread (Ashraf-Ganjouei et al., 2019; Chiang, Levin, Wilde, & Haneef, 2016; Hatton et al., 2020). Structural connectivity in TLE has also been linked to epilepsy duration (Chiang et al., 2016; Owen et al., 2021). Structural network disorganization has been associated with increased cognitive deficits in TLE, and it is more closely associated to changes in cognition than morphological abnormalities (Hatton et al., 2020). Many studies used network connectivity to predict patient outcomes from epilepsy surgery (Bonilha et al., 2006; Kreilkamp, Weber, Richardson, & Keller, 2017; Munsell et al., 2015; Sinha et al., 2021; Taylor et al., 2018), finding that incomplete resection of white matter abnormalities is associated with worse seizure outcomes (Kreilkamp et al., 2017; Sinha et al., 2021). Despite the multitude of TLE studies, it is not clear whether widespread network abnormalities relate to patient outcome in eTLE.

Compared with TLE, less is known about brain network (re)organization in eTLE. One major reason for this knowledge gap is that TLE diagnosis is far more common. Furthermore, TLE is more homogenous in localization comprising just the temporal lobe, and usually the hippocampus. In contrast, eTLE can appear in any part of any other lobe. These factors make group studies in eTLE challenging, and novel approaches are needed to take this heterogeneity of localization into account.

The current study addresses three goals: (a) Develop a method to investigate the network connectivity of the RZ, irrespective of eTLE RZ location. (b) Identify network abnormalities in structural connectivity of the RZ prior to surgery. (c) Determine whether the presence of abnormal RZ connectivity pre-surgery influences post-operative seizure outcome.

## MATERIAL AND METHODS

### Participants

We studied 22 individuals with drug-resistant eTLE who underwent pre-surgical evaluation and subsequent resection, and 29 healthy controls. None had previous neurosurgery. MRI scanning was obtained before and 3–4 months after surgery. All participants were scanned with the same acquisition protocol. Sixteen patients had focal cortical dysplasia, with the remaining patients having gliosis (*n* = 4), cavernoma (*n* = 1), and dysembryoplastic neuroepithelial tumor (*n* = 1). Seizure outcome after surgery was classified with the International League Against Epilepsy (ILAE) (Wieser et al., 2001) seizure outcome scale up to 6 years post-surgery. The last reported ILAE score for each patient was used as their symptom status post-surgery. Post-surgical seizure outcome was defined as the following: ILAE score of 1 or 2 as free of disabling seizures, and ILAE score of 3 or more as non-seizure-free. Outcome groups did not differ in duration of follow-up (*p* = .24, two-tail Mann-Whitney test).

### MRI Acquisition and Preprocessing

T1-weighted (T1_w_) and diffusion-weighted MRI were obtained using a 3T GE Signa HDx scanner equipped with an 8-channel phased array coil. Pre- and post-surgical T1_w_ imaging was performed with an IR-FSPGR acquisition with the following parameters (TE = 3.04 ms, TR = 37.68 s, 170 contiguous, 1.1-mm-thick coronal slices containing 256 × 256 matrix, 0.9375 × 0.9375 mm in-plane resolution). Diffusion MRI was collected using a cardiac triggered single shot EPI acquisition (TE = 73 ms, TR = heart-rate dependent, b-value of 1,200 mm^2^ [*δ* = 21 ms, Δ = 29 ms, using maximum gradient strength of 40 mT m^−1^], 52 directions with 6 B0s. Overall 60 axial slices were collected, each 2.4 mm thick containing 96 × 96 matrix, zero-filled to 128 × 128, 1.875 × 1.875 mm in-plane resolution). All post-operative scans were collected within 12 months after surgery.

All patient pre-operative T1_w_ structural scans underwent the standard *recon-all* pipeline from the FreeSurfer toolbox (version 6.0.1) (Fischl, 2012). We used the Lausanne atlas (Hagmann et al., 2008) to parcellate the brain into 128 regions of interest. Post-operative T1_w_ structural scans then underwent a rigid-body linear registration (6 DOF) using FSL’s FLIRT (Jenkinson, Bannister, Brady, & Smith, 2002; Jenkinson & Smith, 2001) to the corresponding pre-operative T1_w_ output from FreeSurfer. Once the post-operative image was aligned with the pre-operative T1_w_ image, tissue removed during surgery was delineated in the pre-operative space using FSLView. All patient resection masks were drawn manually to account for possible changes in morphometry of the remaining tissue (i.e., sagging and/or shrinking resulting from removal of neighboring supporting structure and/or oedema). Locations and overlap of the resection masks between seizure outcome and all patients is displayed in Supplementary Figure S1.

Patient pre-op and healthy control diffusion-weighted images (DWI) were initially corrected for signal drift (Vos et al., 2017), then eddy current and movement artifacts were corrected using the eddy_correct tool, and b-vectors were rotated accordingly (Jenkinson, Beckmann, Behrens, Woolrich, & Smith, 2012). The DWI were input into DSI studio (version 08.11.2020) and underwent the standard q-space diffeomorphic reconstruction (QSDR) (Yeh & Tseng, 2011) with a diffusion sampling length ratio of 1.25. All reconstructions were aligned and normalized to the ICBM152 template using a linear registration consisting of an affine transformation (12 degrees of freedom) and nonlinear registration using diffeomorphic mapping. Images were then interpolated to a 1.875 mm isotropic resolution using a cubic spline (Yeh et al., 2018).

### dMRI Postprocessing

**Figure 1.**
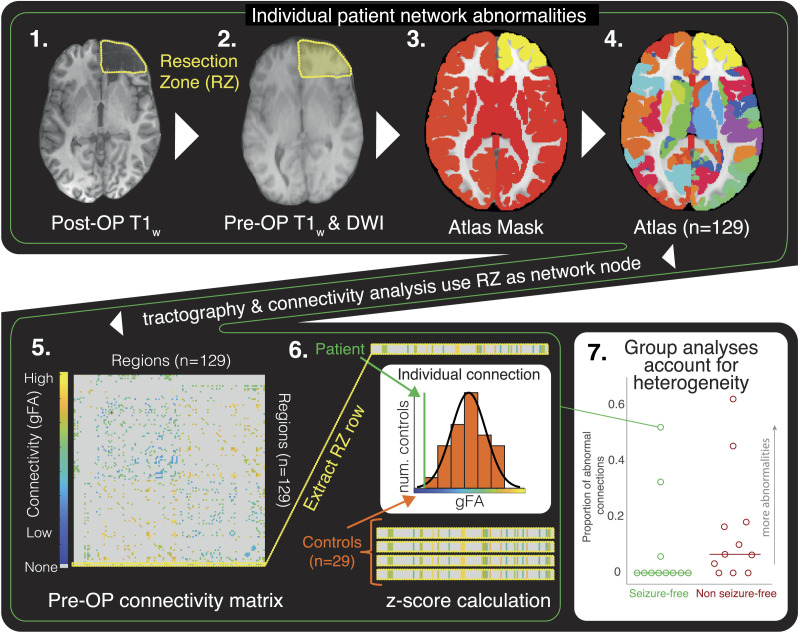
Methods to compare abnormality of the RZ prior to surgery, in a heterogenous eTLE cohort. (Step 1) Resection zone (RZ) delineated in the post-operative T1_w_ scan. (Step 2) The mask is manually drawn in the pre-operative T1_w_ space using the post-operative T1_w_ scan as an anatomical reference. (Steps 3 and 4) Resection mask is imported into the atlas as a new region, removing previous areas in that location. (Step 5) Connectivity matrix is built for each patient and controls to the corresponding specific patient atlas created in Steps 1–4. Tractography streamlines are imported from the HCP842 population average. GFA is used as a measure inferring connectivity strength. (Step 6) Connectivity strengths for connections to the specific resection location for an individual patient are compared with the same RZ area for the control group. The z-score is then generated by comparing the individual patient with the controls as a measure of abnormality. (Step 7) Z-scores are compared between patients to perform group analyses (e.g., of seizure outcome, proportion of abnormal connections).

The following steps are illustrated in Figure 1. Each manually drawn resection mask was imported as an additional region in the Lausanne atlas originally consisting of 128 cortical and subcortical regions (Hagmann et al., 2008) to create a specific atlas for each individual patient (Figure 1, Step 4). We imported the HCP tract template (Yeh et al., 2018) instead of building our own tractography to infer connectivity for four major reasons: (a) Our dMRI data had relatively low angular resolution. (b) Our data had a non-isotropic-sized voxel resolution. (c) Using standardized tracts aided in the replicability of z-scores for normative mapping between patients and controls. (d) Atlas tracts were manually inspected by experienced neuroanatomists. Following from large-scale epilepsy studies reporting widespread reductions of FA in all epilepsies (Hatton et al., 2020), structural connectomes were built using generalized fractional anisotropy (gFA) as a measure of connectivity strength, as gFA provides more robust estimations of anisotropic diffusion at crossing fibers than does fractional anisotropy (Glenn, Helpern, Tabesh, & Jensen, 2015) (Figure 1, Step 5). Streamlines ending in gray matter for regions were counted as connected. Using the HCP842 tracts to infer connectivity coupled with gFA as a measure of connective strength, structural connectomes were built for each patient and for the control group, with each corresponding patient atlas.

To remove the confound of age on gFA, we modeled the effect of age using a robust linear regression model from the control group and regressed out the effect for all patients (*n* = 22) and controls (*n* = 29). After correction, connectivity to a region of interest such as the RZ was analyzed between an individual patient and their controls to generate a z-score representing the relative abnormality pre-surgery. All z-scores were calculated at each individual connection.

### Statistical Testing

As the resection location and size widely varied between patients, we analyzed the proportion of abnormal connections relative to the total number of connections (proportion of abnormality). Following from previous large-scale multicenter research and systematic reviews reporting a reduction of diffusion anisotropy in epilepsy (Hatton et al., 2020; Slinger, Sinke, Braun, & Otte, 2016), abnormal connections were defined as those with substantially reduced gFA, under the 5th percentile (z-score less than −1.96).

**Figure 2.**
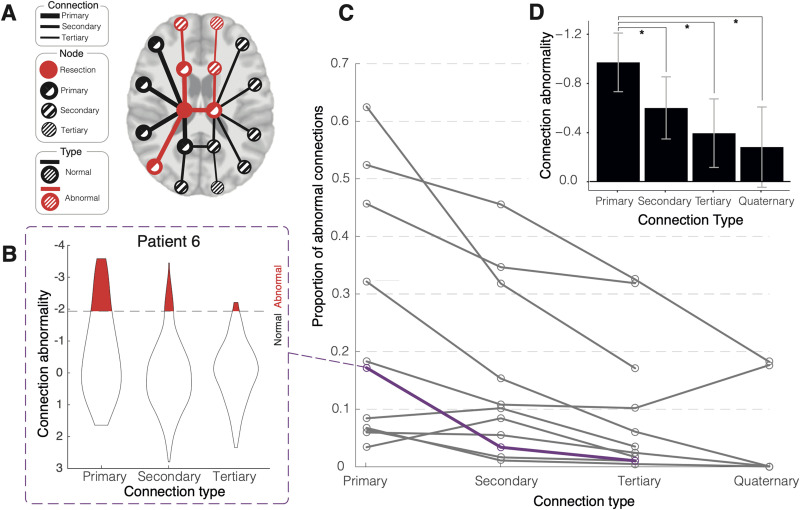
Proportion of abnormal connections decreases with greater topological distance from the resection zone (RZ). (A) Illustration displaying reduced proportional abnormality further from the RZ. In this schematic illustration, three of seven direct RZ connections are abnormal, representing 42.8% of all primary connections. There are five secondary connections (i.e., connections one step removed from the RZ), of which two (40%) are abnormal. Of the three tertiary connections, only one (33.3%) is abnormal. (B) Example violin plot of all z-score connections between differing nodal distances for a single patient highlighted in panel C. Abnormal threshold set at *z* < −1.96. (C) Proportion of abnormal connections for each patient (*n* = 11 had at least one abnormal primary connection) at each nodal distance to RZ. Greater network distance from RZ is associated with fewer abnormalities. The purple line indicates the example patient from panel B. Patients without any abnormal primary connections are omitted for clarity. (D) Hierarchical modeling of the patients in panel C. Connections to primary nodes had significantly more abnormal values compared with subsequent node connections, when accounting for patient as a random intercept, thus confirming the visual impression of panel C (*p* < .05).

To analyze a topological distance effect from the RZ for abnormal connections, patients with at least one single abnormal connection were included in a hierarchical model (Figure 2D). With patient as a random effect, we analyzed the magnitude of connection abnormality against connection type. The connection type was specified as follows: Primary connections were those directly connected to/from the RZ, while secondary connections were those with direct connections to nodes that have a primary connection but not to the RZ. This process was repeated with topological distance to tertiary, and ultimately to quaternary connections that were topologically far from the RZ. All connections were specified as either primary, secondary, tertiary, or quaternary.

We tested whether nodes directly connected to the RZ via an abnormal connection had more abnormal onward connections. We used a paired *t* test to analyze the proportion of abnormal connections between nodes that were directly connected to the resection tissue via a normal (normal node) or abnormal (abnormal node) connection. Lastly, to test whether a group difference in post-operative seizure freedom was evident between patients with one or more abnormal connections to/from the RZ, we used a chi-square test to test differences in seizure outcome between patients who had one or more abnormal connections compared with patients having no abnormal connections to/from the pre-surgical resection area.

## RESULTS

### Connectivity Abnormalities Decrease as Topological Distance Increases for Patients With at Least a Single Abnormal Connection to the RZ

We sought to investigate whether topological distance to/from RZ related to connection abnormality. We therefore categorized network connections as primary, secondary, tertiary, and quaternary. For the patients with one or more abnormal connections to/from the RZ zone (*n* = 11), none had connections beyond quaternary, meaning that the maximum path length to/from the resection was four (Figure 2).

Half of patients (11/22) had one or more abnormal primary connections with the RZ as determined from pre-operative dMRI data. The post-operative MRI is only used to identify the location of the RZ as a node in the network. In an example patient (Figure 2B), there were 41 connections direct to the RZ. Of these 41 direct primary connections, 7 (17%) were abnormal (*z* < −1.96). There were 482 secondary connections, 16 (3%) of which were abnormal (*z* < −1.96). Lastly, there were 409 tertiary connections from the RZ and 4 (1%) were abnormal (*z* < −1.96).

The proportion of abnormal connections decreases as topological distance from RZ increases in 8 out of 11 patients (Figure 2C). We also quantified the relationship between topological distance from resection and connection abnormality in a non-thresholded approach using a hierarchical statistical model. This model used patient as a random effect, to account for patient heterogeneity. With connection type defined as above, our hierarchical analysis (Figure 2D) confirmed significantly greater abnormality in the average primary connection (mean = −0.97, SE = ±0.24), as compared with secondary (mean = −0.60, SE = ±0.25, *p* = .001), tertiary connections (mean = −0.39, SE = ±0.28, *p* = .002), and quaternary connections (mean = −0.28, SE = ±0.33, *p* = .014). These results confirm that the magnitude of connectivity abnormalities decreases as topological distance from the resection increases.

### Abnormalities in Downstream Connections of Nodes Connected to the RZ

**Figure 3.**
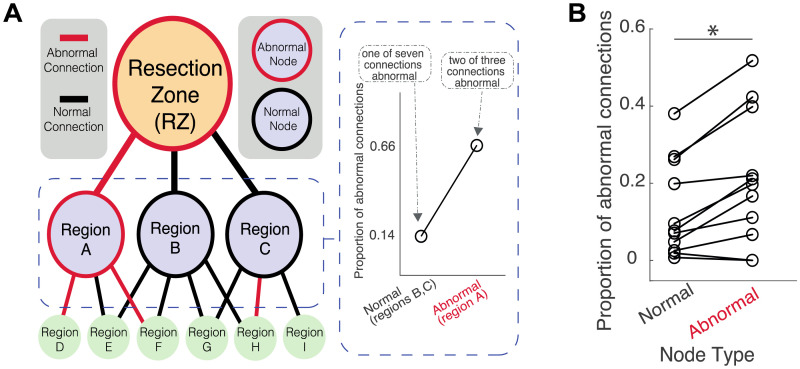
Nodes connected to the RZ have more abnormalities in downstream connections if the upstream connection was abnormal. (A) Illustrative example. Nodes are displayed as circles and lines as connections, abnormal connections and nodes are highlighted in red. A node was defined as abnormal if its upstream connection to the RZ was abnormal. (B) Results from patients with at least one abnormal connection with RZ. There was a higher proportion of abnormal connections if the upstream node had an abnormal connection to the RZ (*p* < .05, paired *t* test).

We analyzed whether regions connected via an upstream abnormal connection to the RZ were more likely to have downstream abnormal connections than regions with a normal upstream connection (Figure 3A). As we were comparing normal and abnormally connected regions to the RZ, we focused on the 11 patients with at least one or more abnormal primary RZ connections.

There were more abnormal connections for nodes connected via an upstream abnormal versus upstream normal connection (*t*(10) = −4.04, *p* = .002). This suggests that regions connected to the RZ via an abnormal connection were more likely to contain abnormal subsequent connections. This finding also suggests a potential spreading effect of abnormal connections from the RZ throughout the wider network.

### Widespread Abnormalities Are More Common in Patients With Poorer Surgical Outcome

**Figure 4.**
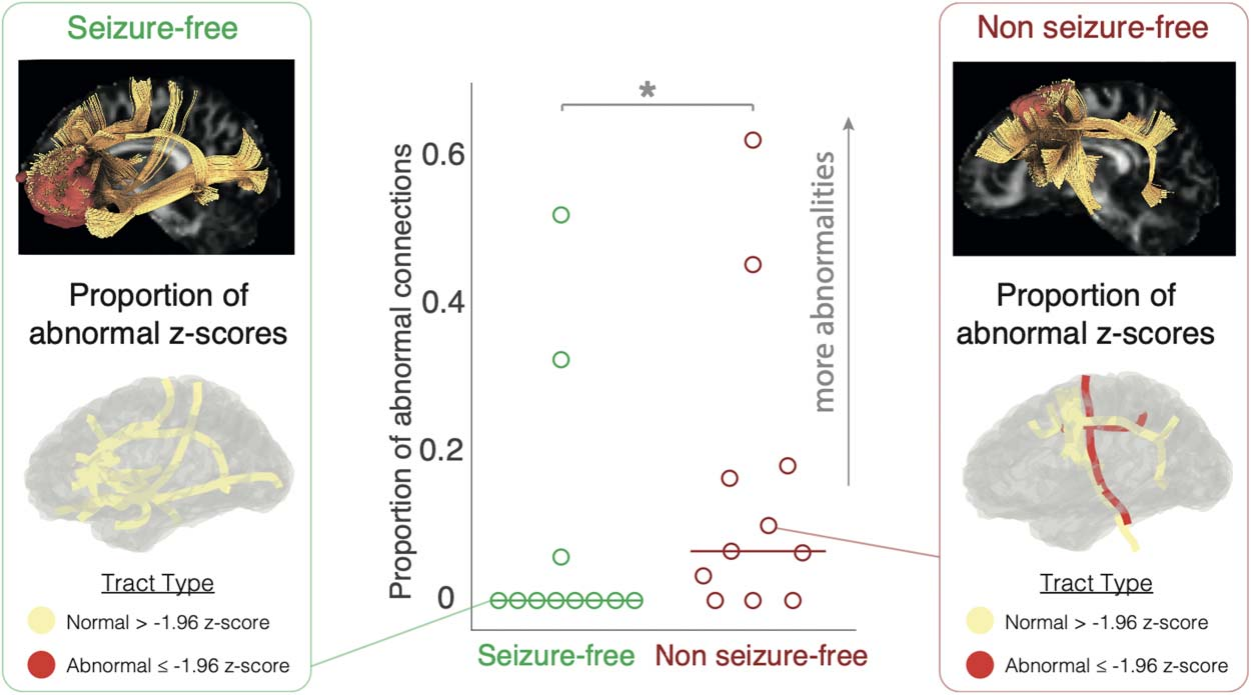
Group difference in resection zone connectivity and seizure outcome. A majority of seizure-free patients did not have a single abnormal connection to/from the RZ (8/11); in contrast, a majority of non-seizure-free patients had one or more abnormal connections (8/11). An example patient (left and right panel) from each group is highlighted to illustrate the difference between groups. The top part of each example displays a sagittal view of the RZ in red, connected tracts in yellow, and a fractional anisotropy map overlaid on the background. The bottom part illustrates on a 3D glass brain surface the same example tracts highlighted as either normal (yellow) or abnormal (red). * *p* < .05.

Our final analysis investigated whether the presence of one or more abnormal connections with the RZ prior to surgery was linked to seizure freedom post-surgery. Three of the 11 seizure-free patients had abnormal connections with the RZ (Figure 4, center panel). In contrast, 8/11 non-seizure-free patients had abnormal connections to the RZ (*X*^2^ = 4.55, *p* = .033) (Figure 4).

## DISCUSSION

We employed a novel method to compare connectivity of the RZ in a heterogenous cohort of eTLE patients. There were three main findings: First, in patients with an abnormal connection at the RZ, the proportion of abnormal connections reduced as topological distance increased from the RZ. Second, regions with an abnormal connection to the RZ had more abnormal subsequent connections than did regions without an abnormal RZ connection. Third, patients with pre-operative abnormal connections with the RZ were less likely to be seizure free after surgery.

Half of the eTLE group had one or more abnormal connections with the RZ. We hypothesized that if the RZ was a central node in a spreading pathological network, the proportion of abnormal connections would reduce the further removed connections were from it. Our first analysis confirmed this hypothesis, showing that proportional reductions were evident in the number of abnormal connections the further removed connections were from the RZ. This suggests that if the RZ has at least one or more abnormal connection, it is a potential epicenter of abnormal connections in the epileptogenic network. These findings concur with studies of structural and functional connectivity, suggesting the epileptogenic zone can affect large-scale networks (Hatton et al., 2020; W. Liu, Yue, Gong, Zhou, & Wu, 2021). Similar findings of widespread network abnormalities have been made in focal epilepsies (Gleichgerrcht et al., 2021; M. Liu, Concha, Lebel, Beaulieu, & Gross, 2012; Owen et al., 2021; Sinha et al., 2021; Tsuda et al., 2018).

Abnormal connections were more likely to occur if they connected to regions with abnormal connections to the RZ. This suggests that abnormality reduces the further removed connections are from the RZ and is more likely to connect from prior abnormal connected regions than from normal connections. With recent findings of widespread abnormal structural networks reported in Alzheimer’s (Lee et al., 2022), Huntington’s (Poudel, Harding, Egan, & Georgiou-Karistianis, 2019), and Parkinson’s disease (Pandya et al., 2019), it would be interesting to investigate whether a similar abnormal spread is present in other diseases.

The finding that patients with abnormal connectivity at the RZ were less likely to be seizure free suggests the existence of a distributed epileptogenic network. Previous studies have similarly found that widespread structural abnormalities in TLE were associated with a poor seizure outcome also (Bonilha et al., 2015; Sinha et al., 2021). Furthermore, widespread functional abnormalities were related to outcome in a recent eTLE study using magnetoencephalographic data (Owen et al., 2023). Taken together, these findings suggest that more widespread structural and functional abnormalities beyond the RZ are associated with poorer post-surgical outcomes.

The findings raise further questions on the nature of abnormality in eTLE networks. First, longitudinal network studies in eTLE would be useful to elucidate dynamic changes in networks, as was recently done in TLE (da Silva et al., 2020; M. Liu et al., 2013). Second, the relationship between abnormal connectivity between regions, and the pathology of those regions, is poorly understood. In TLE a relationship has been demonstrated between nodal atrophy and connectivity abnormality (Horsley et al., 2022); however, this relationship is unexplored in eTLE. Third, although most seizure-free patients had no abnormal connections with the RZ, this does not imply there were no abnormalities *within* the RZ. It would be useful to investigate local white matter tracts within RZ (Chen, Wang, Kopetzky, Butz-Ostendorf, & Kaiser, 2021; Schilling et al., 2022; Shastin et al., 2022). Finally, the postulated epileptogenic zone defined as the RZ may not represent the true epileptogenic foci. It is possible the abnormal connections predominately associated with the post-operative non-seizure-free patients could instead represent a more widespread epileptogenic network, and thus the RZ incompletely covers the full epileptogenic zone (EZ). This mechanism of surgical failure, due to incomplete resection, was recently suggested in another eTLE study (Owen et al., 2023). It would therefore be interesting to investigate this hypothesis further by analyzing the structural connectivity in multifocal epilepsy. It would also be worth in future work to analyze the location of the RZ, particularly if a certain lobe is more prone to abnormal connectivity. Although these findings raise further questions on the nature of abnormal networks in eTLE, we note that the main analysis was conducted on a relatively small sample size, which may impact the reliability and generalizability of the findings. We therefore encourage replication in larger samples in future.

Importing the HCP842 tractography atlas (Yeh et al., 2018) helped standardize and provide accurate white matter connections; furthermore, this technique can be useful in the presence of pathology that may interrupt tractography (da Silva et al., 2020; Horsley et al., 2022; Sinha et al., 2021). In addition, using gFA as a measure of connectivity strength, rather than the more widely used FA, gave a more robust measure of diffusion anisotropy between connections with complex diffusion profiles, particularly for voxels that contain crossing fibers (Glenn et al., 2015). Lastly, inferring connections from streamlines that terminate in two regions, rather than passing through regions, aligns with our hypothesis of connection spread, as each connection would have nonoverlapping streamlines. This research supports a role for dMRI measures to detect changes in eTLE and highlights potential network biomarkers that are predictive of seizure outcome after surgery.

## ACKNOWLEDGMENTS

We thank members of the Computational Neurology, Neuroscience, and Psychiatry Lab (https://www.cnnp-lab.com) for discussions on the analysis and manuscript. We are grateful to the Epilepsy Society for supporting the Epilepsy Society MRI scanner. The authors acknowledge the facilities and scientific and technical assistance of the National Imaging Facility, a National Collaborative Research Infrastructure Strategy (NCRIS) capability, at the Centre for Microscopy, Characterisation, and Analysis, University of Western Australia.

## SUPPORTING INFORMATION

Supporting information for this article is available at https://doi.org/10.1162/netn_a_00327.

## AUTHOR CONTRIBUTIONS

Gerard R. Hall: Conceptualization; Formal analysis; Investigation; Methodology; Project administration; Visualization; Writing – original draft; Writing – review & editing. Frances Hutchings: Formal analysis; Writing – review & editing. Jonathan Horsley: Formal analysis; Validation; Writing – review & editing. Callum M. Simpson: Formal analysis; Validation; Writing – review & editing. Yujiang Wang: Conceptualization; Investigation; Validation; Writing – review & editing. Jane de Tisi: Data curation. Anna Miserocchi: Data curation; Writing – review & editing. Andrew W. McEvoy: Data curation; Writing – review & editing. Sjoerd B. Vos: Data curation; Writing – review & editing. Gavin P. Winston: Data curation; Writing – original draft. John S. Duncan: Data curation; Writing – review & editing. Peter N. Taylor: Conceptualization; Data curation; Funding acquisition; Methodology; Project administration; Resources; Supervision; Validation; Visualization; Writing – review & editing.

## FUNDING INFORMATION

Peter N. Taylor, UK Research and Innovation (https://dx.doi.org/10.13039/100014013), Award ID: MR/T04294X/1. Yujiang Wang, UK Research and Innovation (https://dx.doi.org/10.13039/100014013), Award ID: MR/V026569/1. Gavin P. Winston, Medical Research Council (https://dx.doi.org/10.13039/100012891), Award ID: G0802012. Gavin P. Winston, Medical Research Charities Group (https://dx.doi.org/10.13039/100012891), Award ID: MR/M00841X/1. Sjoerd B. Vos, NCRIS. Jane de Tisi, National Institute for Health and Care Research (https://dx.doi.org/10.13039/501100000272). Anna Miserocchi, National Institute for Health and Care Research (https://dx.doi.org/10.13039/501100000272). Andrew W. McEvoy, National Institute for Health and Care Research (https://dx.doi.org/10.13039/501100000272). Gavin P. Winston, National Institute for Health and Care Research (https://dx.doi.org/10.13039/501100000272). John S. Duncan, National Institute for Health and Care Research (https://dx.doi.org/10.13039/501100000272). Callum M. Simpson, EPSRC. Jonathan Horsley, EPSRC.

## TECHNICAL TERMS

Focal epilepsy: Major type of epilepsy where symptoms are generated from a particular location of the brain.
Seizure onset zone (SOZ): The true location(s) in the brain where seizures originate and from where they can subsequently spread.
Resection zone (RZ): The location of the brain that has been removed by surgical resection.
Extra temporal lobe epilepsy (eTLE): A subtype of focal epilepsy that is situated in regions other than the temporal lobe.
ILAE score: A score that describes post-surgical seizure recurrence, ranging from 1 (no seizures or auras) to 6 (significant increase in seizures and auras after surgery).
Tractography: A technique that models diffusion MRI data into a three-dimensional representation of white matter fiber connections.
Generalized fractional anisotropy (gFA): A measure of the diffusion shape in terms of anisotropy. It is similar to fractional anisotropy but calculated using the orientation distribution function.
Abnormal connection: A connection that has gFA lower than the 5th percentile of the control distribution for that connection.
Epileptogenic zone (EZ): Often encompassing the seizure onset zone, EZ refers to the brain areas indispensable for the generation of seizures.
