# Supplementary material for: Epileptogenic networks in extra temporal lobe epilepsy
Source: Netw Neurosci. 2023 Dec 22;7(4):1351–62. doi: 10.1162/netn_a_00327 (PMC10631792; doi:10.1162/netn_a_00327)
Supplement: Supplementary file 1 [file netn-7-4-1351-s001.docx]

**Supplementary Figures**

Figure S1: Location and overlap of the RZ for the seizure free, non-seizure free and both groups on sequential coronal slices (top right, sagittal reference is included).

Figure S2. (Top) Total volume for each mask drawer on a randomly selected subset of 8 patients. Each data point represents an individual patient. Dashed line displays the strength of the spearman’s correlation. (Bottom) Histogram showing the Sørensen-Dice similarity coefficient between the two raters for the subset of patients.

Figure S3: Displays the same statistical analysis of Figure 2, 3 and 4 from the main text, however at a finer grained parcellation of the Lausanne atlas containing 463 regions (Hagmann et al., 2008). (Top) Shown on the line-graph are 14 of the 22 patients that had one or more abnormal primary connections direct to/from the RZ. The fine grain atlas showed similar significant reductions in abnormal connections (z-score <-1.96) between Primary to; Secondary (*p*= 0.04), Tertiary (*p*= 0.002) and quaternary (*p*= 0.001) nodes. (Bottom, Left) Of the 14 patients with one or more abnormal connections, paired t-test reported nodes connected via a direct abnormal connection to the RZ had a higher proportion of subsequent abnormal connections compared to normal connected nodes t(13)= -4.58, *p*< .001. (Bottom, Right) For all patients (n=22), when analysing the presence of an abnormal connection at the primary node against post-operative seizure freedom, again a similar trend was evident compared to the main analysis, however no statistical significance was present at the higher parcellation (*X^2^*= 3.14, *p*= 0.076) (* = *p*≤ 0.05, n.s = non-significant).

Figure S4: Relationship between the percentage of abnormal primary connections at the RZ, and the mask volume (mm^3^). A spearman’s correlation analysis reported a positive correlation (r= 0.36), although this was insignificant (*p*= 0.10).
